# Growth differentiation factor 15 induces growth and metastasis of human liver cancer stem-like cells via AKT/GSK-3β/β-catenin signaling

**DOI:** 10.18632/oncotarget.15216

**Published:** 2017-02-09

**Authors:** Qiong Xu, Hai-Xu Xu, Jin-Ping Li, Song Wang, Zheng Fu, Jing Jia, Li Wang, Zhi-Feng Zhu, Rong Lu, Zhi Yao

**Affiliations:** ^1^ Department of Immunology, Tianjin Medical University, Tianjin, China; ^2^ Tianjin Kangzhe Pharmaceutical Company, Ltd., Tianjin, China

**Keywords:** cancer stem cells, hepatocellular carcinoma, GDF15, metastasis, tumorigenesis

## Abstract

Cancer stem cells in liver cancer are thought to be responsible for tumor recurrence and metastasis. However, the factors that mediate this mechanism have yet to be completely elucidated. In this study, we isolated CD13^+^CD44^+^ sphere cells (SCs) derived from liver cancer tissues and SK-Hep-1 cells, which possessed cancer stem cell-like properties. Through cytokine array analysis, growth differentiation factor 15 (GDF15) was significantly increased in SCs. Clinical data showed GDF15 was overexpressed in liver cancer tissues and was positively related to pathological grading. GDF15 knockdown significantly inhibited the growth and metastasis of SCs through AKT/GSK-3β/β-catenin pathway suppression. Moreover, a PI3K inhibitor LY294002 inhibited AKT/GSK-3β/β-catenin pathway activated by GDF15 and attenuated GDF15-induced proliferation, colony formation and invasion of SCs. Conclusion: Our studies suggest that CD13^+^CD44^+^ SCs may represent a subset of LCSCs. GDF15 promotes the growth and metastasis of SCs by activating AKT/GSK-3β/β-catenin signaling pathway. Promisingly, GDF15 could be considered as a potential therapeutic target in liver cancer.

## INTRODUCTION

Hepatocellular carcinoma (HCC) is the fifth most common malignancy worldwide, and the third leading cause of cancer-related deaths [1]. The worldwide incidence of HCC varies according to the prevalence of hepatitis B (HBV) and hepatitis C (HCV) infection. Chronic HCV infection is the leading cause of HCC in developed countries whereas HBV infection is the leading cause in the majority of developing countries. Other risk factors that are more common in western countries include obesity, diabetes, cirrhosis related to heavy alcohol consumption and others [2, 3]. Although advances in liver cancer detection and treatment have increased the possibility of curing the disease at early stages, unfortunately, the prognosis of most HCC patients is still poor, due to high rates of tumor recurrence and metastasis [4]. It is imperative to further investigate the molecular mechanisms of HCC recurrence and metastasis for monitoring the progress of liver cancer therapy and for developing effective therapeutic strategies.

Various factors such as cancer stem cell (CSC) and epithelial-mesenchymal transition (EMT) contribute to tumor recurrence and metastasis [5, 6]. Recent studies suggest that a primary cause of HCC relapse and metastasis is the persistence of liver cancer stem cells (LCSCs), which are highly tumorigenic, metastatic and resistant to chemotherapy [7, 8]. Yamashita et al [9] suggested that HCC growth and invasiveness were controlled by a subset of EpCAM^+^ CSCs. Most recently, the effect of CD90^+^ CSCs on enhanced cell motility of EpCAM^+^ cancer stem cells was mediated, at least in part, via activation of TGF-β signaling by CD90^+^ CSCs [10]. Similar evidence demonstrated that liver-specific Notch signaling activation was associated with tumorigenesis and metastasis of LCSCs [11]. All these studies demonstrate that LCSCs are a pivotal therapeutic target in the eradication of HCC. While several possible signaling molecules related to growth and metastasis of LCSCs have been reported, the factors that mediate this role have not yet been completely elucidated. Further studies are needed to clarify the molecular mechanism of LCSCs in promoting metastasis and relapse of HCC.

In the current study, we successfully isolated and expanded CD13^+^CD44^+^ sphere cells (SCs) derived from clinical HCC samples and SK-Hep-1 cells, which demonstrated highly tumorigenic, chemo-resistant and metastatic potential. Human cytokine antibody arrays were applied to screen for cytokines which may mediate the growth and metastasis of LCSCs. Colony formation and transwell assay were performed to investigate whether GDF15 is involved in the proliferation and invasion of LCSCs *in vitro*. Subcutaneous xenotransplanted tumor and pulmonary metastatic animal models of LCSCs were established to further study the role of GDF15 in tumorigenesis and metastasis of LCSCs *in vivo*. Finally, the molecular mechanisms of GDF15 involved in tumorigenesis and metastasis of LCSCs were explored.

## RESULTS

### Sphere cells derived from human HCC samples and SK-Hep-1 cells represent a group of CD13^+^CD44^+^ cells, which preferentially express stem cell-associated genes

**Figure 1:**
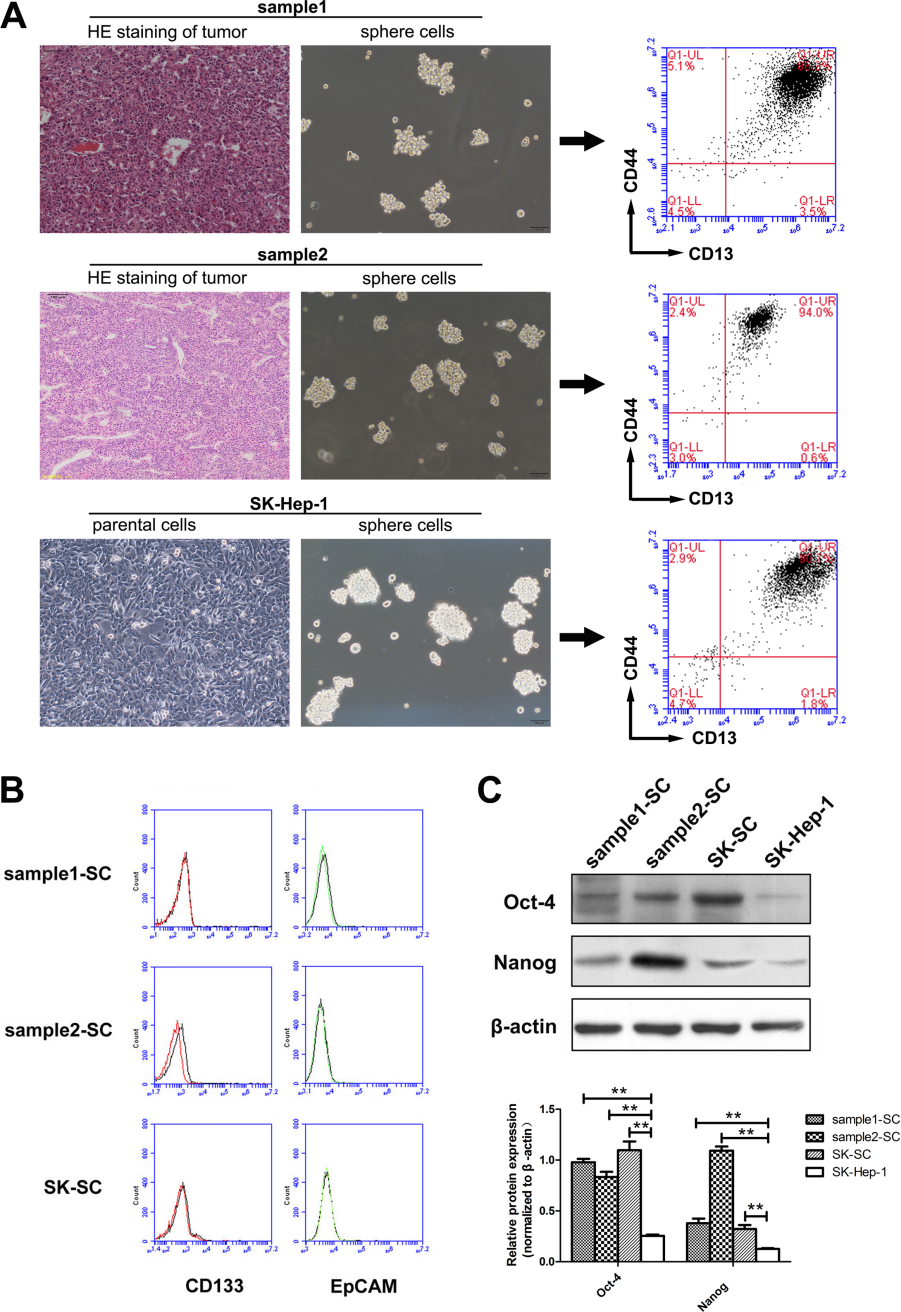
SCs derived from two HCC samples and SK-Hep-1 cells represent a group of CD13+CD44+ cells, which preferentially express stem cell-associated genes (**A**) Formation and amplification of SCs in clinical HCC samples and SK-Hep-1 cells. HE staining of tumors derived from two clinical samples revealed hepatocellular carcinoma. Clinical samples digested with collagenase and SK-Hep-1 cells were cultured in SFM. A minority of floating cells formed larger spheres of 100 to 200 cells after about 14 days. Scale bar: 100 μm. The expression of CD13 and CD44 in three groups of SCs was analyzed by flow cytometry. (**B**) The expression of CD133 and EpCAM in three groups of SCs was analyzed by flow cytometry. (**C**) The expression levels of stem cell-associated proteins Oct-4 and Nanog in SCs and SK-Hep-1 cells were detected by western blot analysis. Relative protein intensities were detected by Image J software. β-Actin was used as a loading control. **: compared with SK-Hep-1 cells, *P < 0.01*.

Among the 40 HCC samples and four HCC cell lines (SK-Hep-1, Hep3B, Bel-7402 and SMMC-7721), SCs were established and cultured successfully in two liver cancer samples and SK-Hep-1 cells. The majority of tumor cells cultured in serum-free medium (SFM) died, with a few floating cells forming spheres of 3 to 5 cells. After 10 to 14 days, large tumor spheres with bright appearance and sharp edges were found, each composed of 100 to 200 cells (Figure 1A). During the formation of primary tumor spheres, cells were collected by gravity and dissociated enzymatically for amplification. Meanwhile, we analyzed the expression of several markers proposed in the literature to identify LCSCs in the three groups of SCs. Flow cytometry analysis demonstrated that all three groups of SCs co-expressed CD13 and CD44 (Figure 1A), instead of CD133 and EpCAM (Figure 1B). These data indicate that SCs derived from human HCC samples and SK-Hep-1 cells may represent a group of CD13^+^CD44^+^ cells. To further determine whether SCs possess stem cell-like properties, we examined the expression levels of a few stem cell-associated genes underlying self-renewal and differentiation of CSCs. We found that the expression of Oct-4 and Nanog in these SCs was much higher than in SK-Hep-1 cells (Figure 1C).

### SCs display higher chemo-resistance and higher clonogenicity

**Figure 2:**
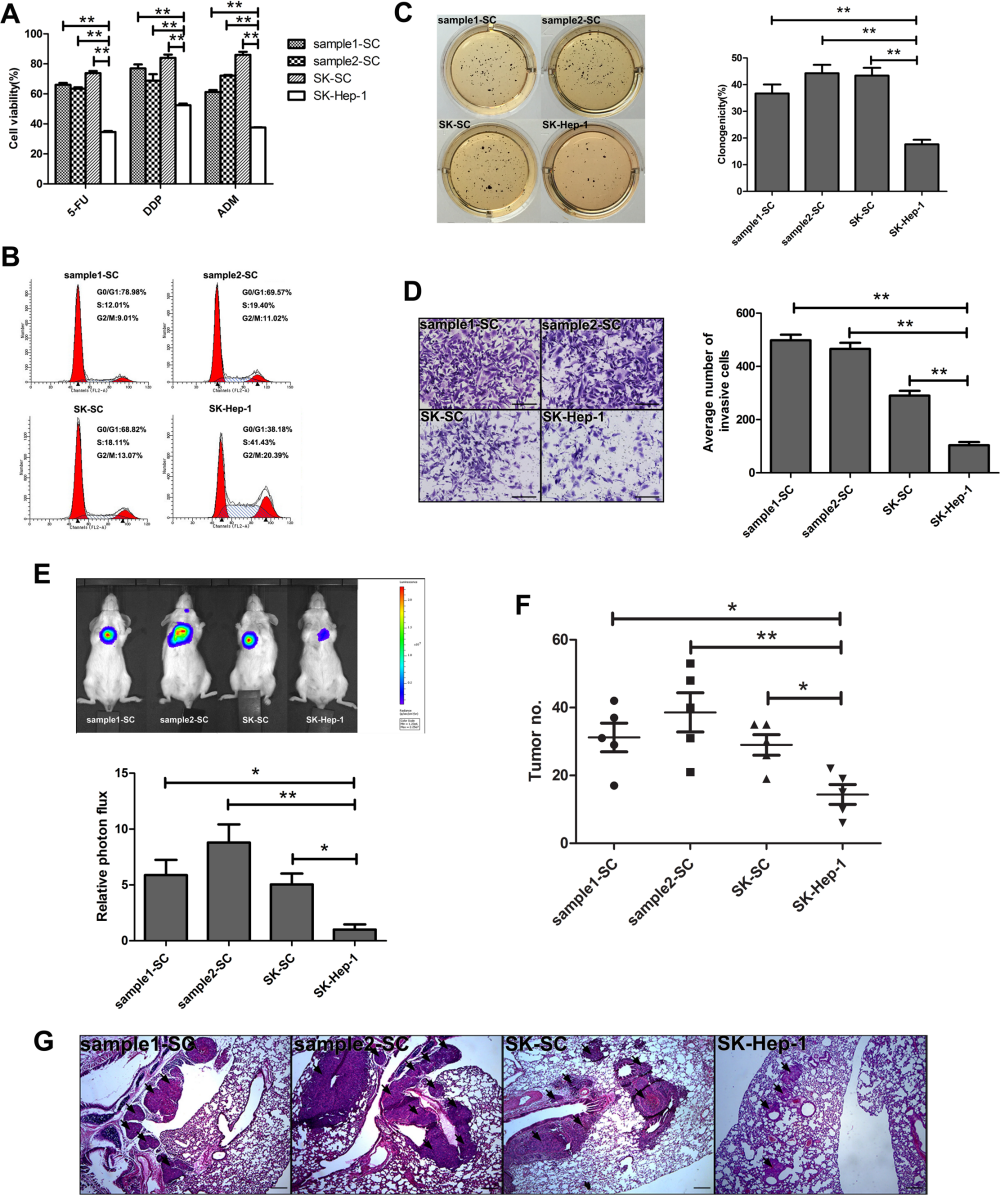
SCs derived from two HCC samples and SK-Hep-1 cells exhibit cancer stem cell-like properties (**A**) Resistance to chemotherapeutic drugs in SCs and SK-Hep-1 cells. Cells were treated with 5-FU, DDP and ADM for 48 hours. Cell viability was measured by MTS and calculated using the following formula: Cell viability (%) = (absorbance of treated cells)/(absorbance of untreated cells)×100%. The data are shown as the means ± SD (*n* = 3). **: compared with SK-Hep-1 cells, *P < 0.01*. (**B**) Cell cycle distributions of SCs and SK-Hep-1 cells were detected by flow cytometry. (**C**) The clonogenicity of SCs and SK-Hep-1 cells was detected by colony formation assay. Colonies were labeled with MTT. The number of colonies was counted. The data are shown as the means ± SD (*n* = 3). **: compared with SK-Hep-1 cells, *P < 0.01*. (**D**) The invasive ability of SCs and SK-Hep-1 cells was detected by transwell assay. Images were obtained after 48 hours of invasion, and the number of invaded cells was counted in three different fields per sample. Scale bar: 50 μm. **: compared with SK-Hep-1 cells, *P < 0.01*. (**E** and **F**) The pulmonary metastasis of SCs and SK-Hep-1 cells was monitored by bioluminescence imaging *in vivo*. The growth of pulmonary metastasis was observed using whole-body fluorescent imaging system. Relative photon flux of the four groups was calculated and tumor number was measured. The data are shown as the means ± SD (*n* = 5). *: compared with SK-Hep-1 cells, *P < 0.05*; **: compared with SK-Hep-1 cells, *P < 0.01*. (**G**) Representative images of HE staining of lungs derived from the four groups after 4 weeks of cell injection. Scale bar: 100 μm.

One of the important properties of CSCs is their resistance to conventional chemotherapy. Therefore, we used three conventional chemotherapeutic drugs to evaluate the chemo-resistance in SCs and SK-Hep-1 cells. As shown in Figure 2A, the survival rates of sample1-SCs, sample2-SCs and SK-SCs were higher under the treatment of 5-Fluorouracil (5-FU), cisplatin (DDP) and adriamycin (ADM), compared with SK-Hep-1 cells. Meanwhile, classical multidrug resistance is attributed to an elevated expression of ATP-dependent drug efflux pumps that belong to ATP-binding cassette (ABC) transporters such as multidrug resistance gene-1 (MDR1), multidrug resistance associated proteins 1 (MRP1) and breast cancer resistance protein (BCRP/ABCG2) [12]. Our results revealed that the expression of MDR1, MRP1 and ABCG2 was significantly increased in three groups of SCs (Supplementary Figure 1). These data suggest that some ABC transporters involved in multidrug resistance of SCs lead to higher tolerance to chemotherapeutic drugs.

Stem cells are considered to be slow-cycling cells. The cell cycle distribution of SCs and SK-Hep-1 cells is shown in Figure 2B. Compared with SK-Hep-1 cells, the cell cycles of the sample1-SCs, sample2-SCs and SK-SCs were altered with marked increase in the G0/G1 phase and decrease in S and G2/M phase. Cell cycle analysis demonstrated that SCs grew at slower rates than SK-Hep-1 cells. However, three groups of SCs showed remarkably higher colony formation efficiency than SK-Hep-1 cells (Figure 2C). All these results suggest that SCs are highly resistant to chemotherapy and display extensive capacities for proliferation and self-renewal as a possible CSC subset.

### SCs exhibit high tumorigenicity and metastatic potential *in vivo*

To determine whether SCs possess higher tumorigenicity than SK-Hep-1 cells *in vivo*, we conducted tumor formation experiments using sample1-SCs, sample2-SCs, SK-SCs and SK-Hep-1 cells. Various numbers of SCs and SK-Hep-1 cells, ranging from 100 to 10,000, were injected subcutaneously into NOD/SCID mice with weekly monitoring for tumor development. An apparent difference in tumor incidence was observed between SCs and SK-Hep-1 cells (Table 1). As few as 100 sample1-SCs, 100 sample2-SCs and 250 SK-SCs were sufficient to induce tumor formation in immunodeficient mice, and 100% tumor development was observed in 1,000 sample1-SCs, 500 sample2-SCs and 1,000 SK-SCs, respectively. In contrast, injection of up to 5,000 SK-Hep-1 cells resulted in tumor formation at a lower efficiency (1 of 4 injected mice) than in SCs. Furthermore, we investigated the invasive ability of SCs *in vitro* using transwell assay. Compared with SK-Hep-1 cells, SCs showed stronger invasive ability (Figure 2D). To determine the metastatic potential of SCs *in vivo*, we transduced three groups of SCs and SK-Hep-1 cells with luciferase-expressing lentiviral vectors and injected them into NOD/SCID mice via the tail vein, respectively. The original transduction efficiency of luciferase-expressing SCs and SK-Hep-1 cells is similar (Supplementary Figure 2). The luciferase signals of the three groups of SCs were significantly higher than that of SK-Hep-1 cells, especially sample2-SCs (Figure 2E). The number of lung metastases was markedly increased in SCs (Figure 2F). Histological analysis further confirmed that mice injected with SCs manifested more and bigger metastatic tumors of the lung (Figure 2G). The foregoing results suggest that three groups of SCs manifest higher tumorigenicity and pulmonary metastasis.

**Table 1:** Tumorigenicity of sphere cells in NOD/SCID mice

Cells	1 × 10^2^	2.5 × 10^2^	5 × 10^2^	1 × 10^3^	2.5 × 10^3^	5 × 10^3^	1 × 10^4^
sample1-SC	3/5(60%)	2/4(50%)	3/4(75%)	4/4(100%)	4/4(100%)	4/4(100%)	4/4(100%)
sample2-SC	3/5(60%)	3/4(75%)	4/4(100%)	4/4(100%)	4/4(100%)	4/4(100%)	4/4(100%)
SK-SC	0/5	2/4(50%)	3/4(75%)	4/4(100%)	4/4(100%)	4/4(100%)	4/4(100%)
SK-Hep-1	0/5	0/4	0/4	0/4	0/4	1/4(25%)	2/4(50%)

### GDF15 is overexpressed in SCs derived from clinical HCC samples and SK-Hep-1 cells

**Figure 3:**
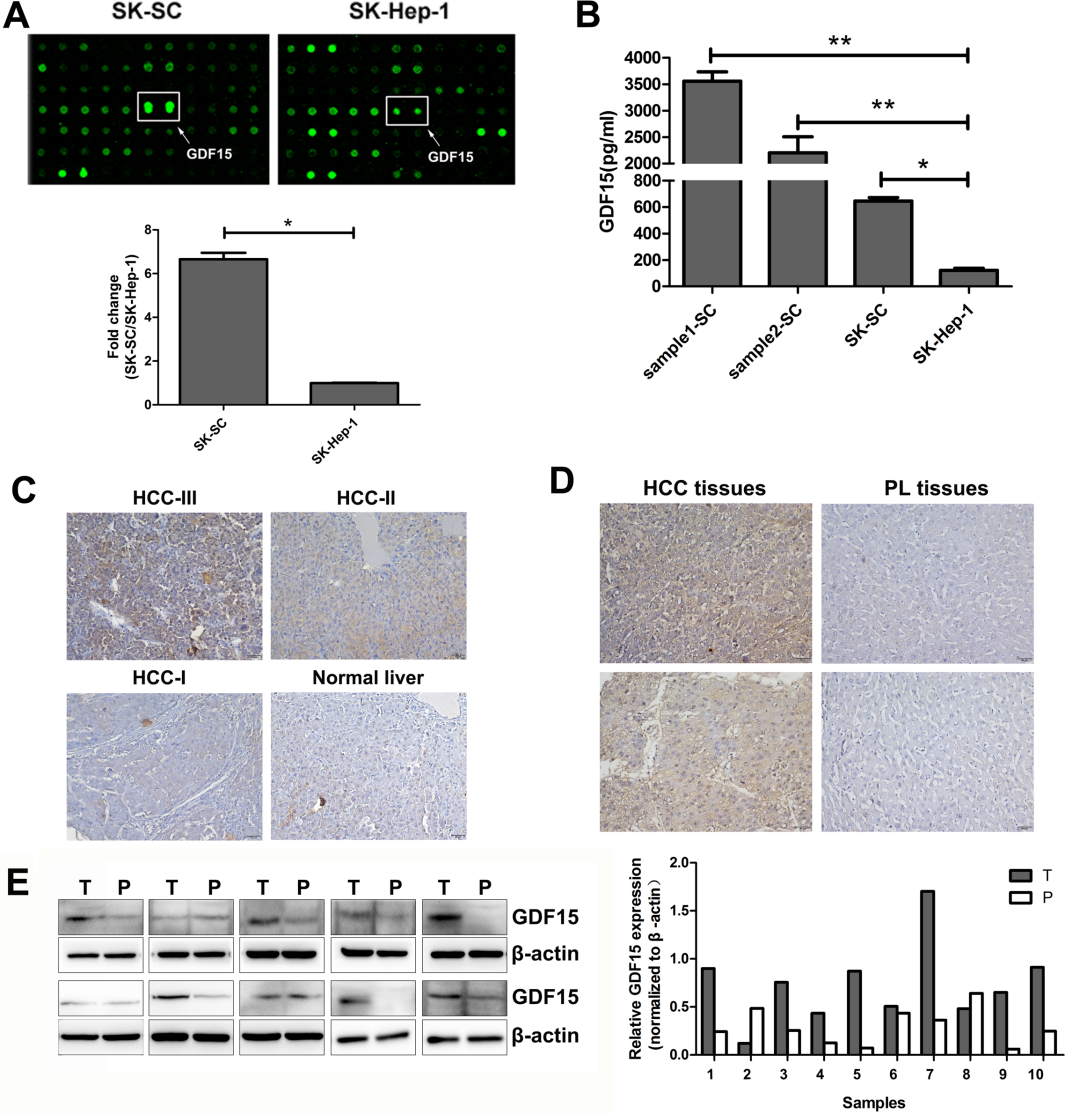
GDF15 is highly expressed in three groups of SCs and HCC tissues (**A**) Cytokine antibody arrays of cytokine expression in culture supernatants of SK-SC and SK-Hep1 cells. GDF15 expression in SK-SCs and SK-Hep-1 cells was detected by cytokine antibody array. The data are shown as the means ± SD (*n* = 3). *: compared with SK-Hep-1 cells, *P < 0.05*. (**B**) Sample1-SCs, sample2-SCs, SK-SCs and SK-Hep-1 cells were incubated in serum-free media for 48 hours. GDF15 concentration was determined using ELISA in media from cells. The data are shown as the means ± SD (*n* = 3). *: compared with SK-Hep-1 cells, *P < 0.05*; **: compared with SK-Hep-1 cells, *P < 0.01*. (**C**) A HCC tissue array consisting of HCC samples with different grades of malignancy and normal liver samples was stained for GDF15. Representative results are shown. Scale bar: 50 μm. (**D**) GDF15 expression in HCC and PL tissues from two patients was determined by IHC. Scale bar: 50 μm. (**E**) GDF15 protein expression in HCC (T) and PL (P) lysates was determined by western blot analysis. Relative protein intensities were detected by Image J software. β-Actin was used as a loading control.

To identify the cytokines secreted by LCSCs that drive tumor recurrence and metastasis, the cytokine profiles of culture medium (CM) containing SK-SCs and SK-Hep-1 cells were analyzed using RayBio Human Cytokine Antibody Array. We found that the expression of GDF15 in SK-SCs was higher than in SK-Hep-1 cells (Figure 3A). ELISA further confirmed the increased GDF15 levels in the CM of the three groups of SCs, especially in the CM of sample1-SCs and sample2-SCs (Figure 3B).

### GDF15 expression is increased in HCC and shows a significant correlation with clinicopathologic characterization

Elevated expression of GDF15 was previously reported in serum and tumor samples of HCC patients [11]. To obtain further evidence that GDF15 expression is increased in HCC, we performed immunohistochemistry (IHC) on a human HCC tissue assay. HCC tissues and normal liver tissues were used for IHC with an antibody against GDF15. As shown in Table 2, 22 of 30 (73.3%) tumor tissues showed GDF15-positive staining, as compared to five normal liver tissue samples, none of which stained positive for GDF15. The representative data are shown in Figure 3C. GDF15 expression was increased in moderate (HCC-II) or severe (HCC-III) HCC, but not in mild (HCC-I) HCC and normal liver tissues. In addition, ten pairs of HCC and paracarcinomatous liver (PL) tissues were prepared from the same patient to eliminate the effects of differences in the genetic background. IHC and western blotting were performed. GDF15 expression was elevated in 7 of 10 (70%) HCC samples (Figure 3D and 3E). These results suggest that GDF15 is overexpressed in a part of HCC tissues.

**Table 2:** IHC analysis of GDF15 on HCC tissue array

	GDF15			
	Negative		Positive	
	N	%	N	%
HCC tissues (*n* = 30)	8	26.7	22	73.3*
Normal liver tissues (*n* = 5)	5	100	0	0

* *P* = 0.004.

To explore any correlation between GDF15 expression and clinicopathologic characteristics of HCC, we detected the expression of GDF15 in HCC samples using IHC. The elevated GDF15 expression showed a significant correlation with pathological grading. However, no correlation between age, gender and TNM stage was detected (Table 3).

**Table 3:** Correlation between GDF15 expression and clinicopathologic characteristic of HCC patients

Characteristic	Number	GDF15 expression		*P*
		Low	High	
Age(yr)				
≤ 50	18	6	12	0.842
> 50	22	8	14	
Gender				
Male	27	10	17	0.697
Female	13	4	9	
Pathological grading				
I/II	20	10	10	0.047
III	20	4	16	
TNM stage				
I/II	17	8	9	0.169
III	23	6	17	

### GDF15 knockdown suppresses the proliferation and colony formation ability of SCs, but has no effect on chemo-resistance

**Figure 4:**
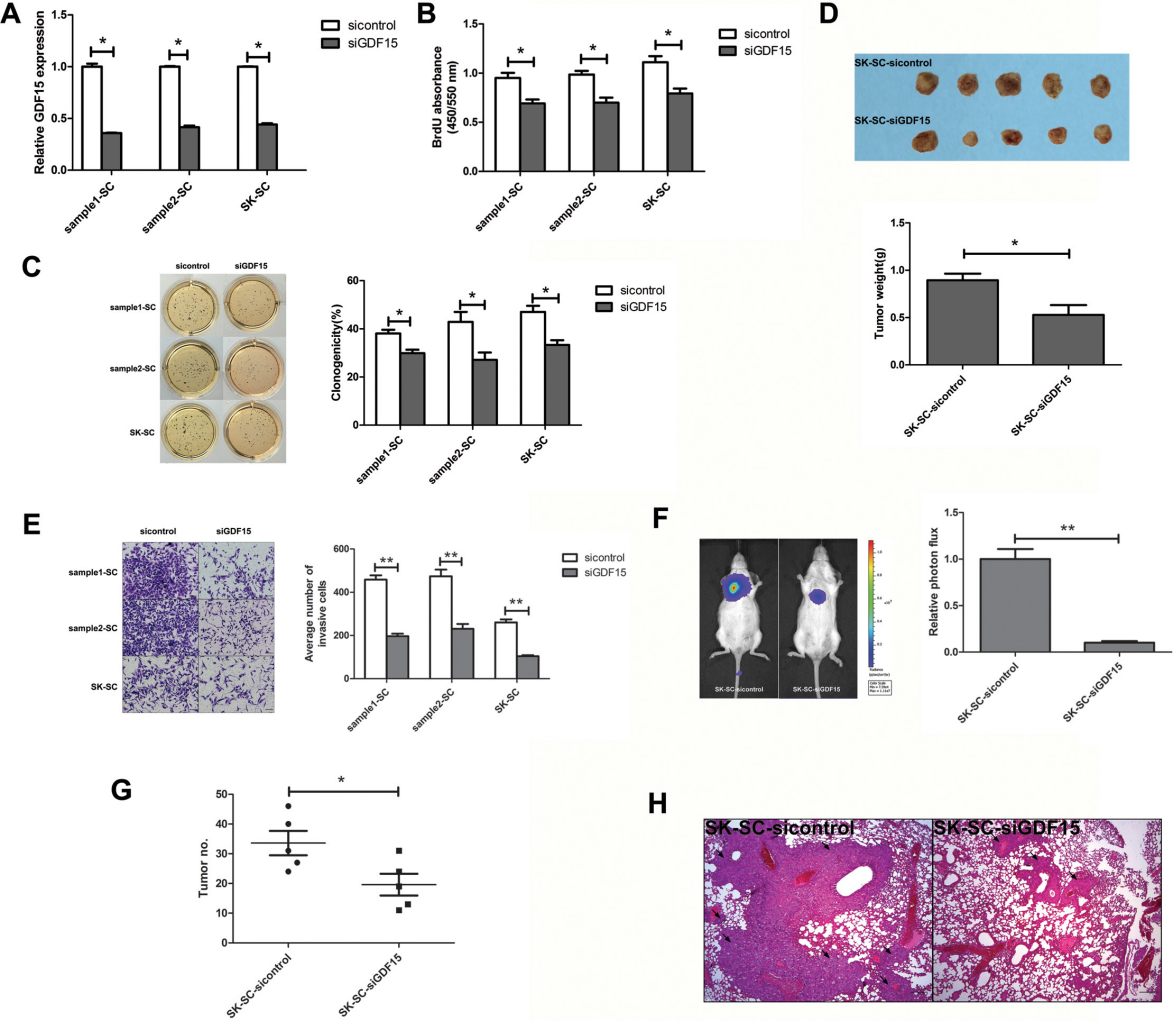
GDF15 knockdown inhibits the growth and metastasis of SCs *in vitro* and *in vivo* (**A**) GDF15 concentration was determined by ELISA in culture supernatants derived from SCs transfected with siGDF15 and sicontrol, respectively. The data are shown as the means ± SD (*n* = 3). *: compared with control group, *P < 0.05*. (**B**) The proliferation of GDF15 knockdown SCs was detected by BrdU. Values represent the average absorbance in five duplicates per group. *: compared with control group, *P < 0.05*. (**C**) The clonogenicity of GDF15 knockdown SCs was detected by colony formation assay. Colonies were labeled with MTT. The number of colonies was counted. The data are shown as the means ± SD (*n* = 3). *: compared with control group, *P < 0.05*. (**D**) Tumorigenicity of SK-SCs transfected with siGDF15 and sicontrol. Cells (1 × 10^6^) were injected subcutaneously into the flanks of recipient NOD/SCID mice (*n* = 5). Animals were sacrificed when tumor nodules were identified on the body surface of mice. Tumor weight was weighed. The data are shown as the means ± SD. *: compared with control group, *P < 0.05*. (**E**) The invasive ability of GDF15 knockdown SCs was detected by transwell assay. Images were obtained after 48 hours of invasion, and the number of invaded cells was counted in three different fields per sample. The data are shown as the means ± SD (*n* = 3). Scale bar: 50 μm. **: compared with control group, *P < 0.01*. (**F** and **G**) The pulmonary metastasis of SK-SCs transfected with siGDF15 and sicontrol was monitored by bioluminescence imaging *in vivo*. The growth of pulmonary metastasis was observed using whole-body fluorescent imaging system. Relative photon flux of the two groups was calculated and tumor number was measured. The data are shown as the means ± SD (*n* = 5). *: compared with control group, *P < 0.05*; **: compared with control group, *P < 0.01*. (**H**) Representative images of HE staining of lungs derived from both groups after 8 weeks of cell injection. Scale bar: 100 μm.

To determine the significance of GDF15 in SCs, we suppressed the expression of GDF15 in SCs using lentiviral GDF15 shRNA. GDF15 expression was inhibited in cells transfected with GDF15-shRNA (shGDF15) in contrast to cells transfected with control shRNA (shcontrol), by ELISA analysis (Figure 4A). To investigate the effects of GDF15 knockdown on SC proliferation and colony formation ability, BrdU and colony formation assays were performed. BrdU assay showed that for three groups of SCs proliferation ability was significantly inhibited in the GDF15 knockdown group compared with the control group (Figure 4B). Consistently with the BrdU assay results, GDF15 knockdown decreased SCs colony formation (Figure 4C). Thus, GDF15 knockdown significantly attenuates SC proliferation and colony formation ability, but no significant differences were found between GDF15 knockdown and control groups in terms of drug resistance (Supplementary Figure 3).

### GDF15 knockdown inhibits the tumorigenesis and metastasis of SCs

The effect of GDF15 on xenograft tumor growth was investigated *in vivo*. SK-SCs transfected with shGDF15 and shcontrol were injected subcutaneously into the flanks of recipient NOD/SCID mice. After 40 days, tumor incidence was detected in both the groups, and the volume and weight of the finally resected tumors in the GDF15 knockdown groups were significantly lower than those of the control group (Figure 4D). We used transwell assay to compare changes in invasion ability before and after shGDF15 vector transfection in three groups of SCs. The results demonstrated that GDF15 knockdown decreased SCs invasion (Figure 4E). Moreover, we further investigated the effects of GDF15 on tumor metastasis *in vivo* using a mouse model of lung metastasis. Luciferase-expressing SK-SCs were transfected with shGDF15 and shcontrol, and injected intravenously into NOD/SCID mice. As shown in Figure 4F and 4G, GDF15 knockdown in SK-SCs significantly inhibited lung metastasis. Furthermore, HE staining of lung tissue confirmed that mice injected with GDF15 knockdown SK-SCs showed fewer and smaller pulmonary metastases (Figure 4H).

**Figure 5:**
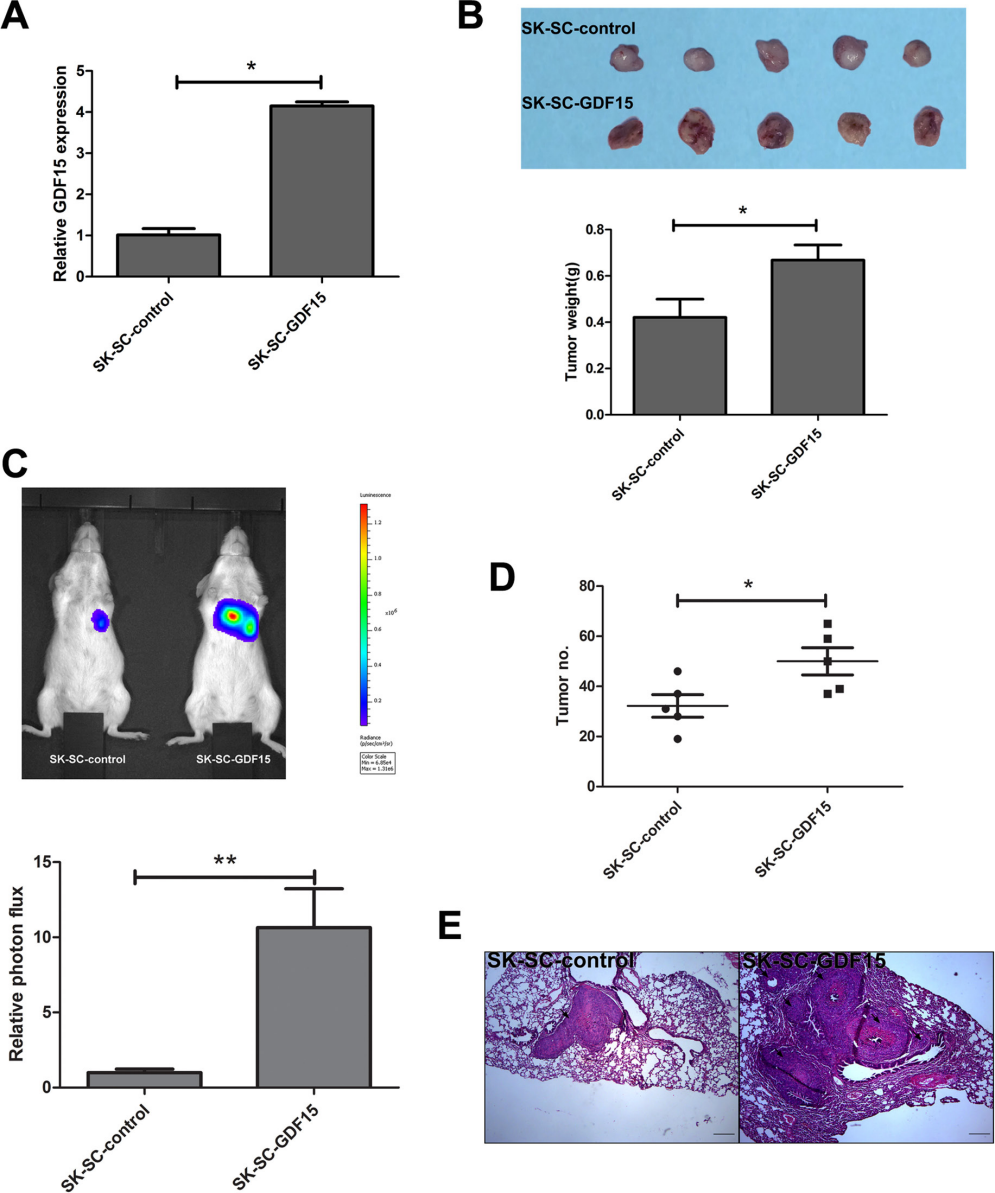
GDF15 overexpression promotes the tumorigenesis and metastasis of SCs *in vivo* (**A**) GDF15 concentration was determined by ELISA in culture supernatants of SCs transfected with GDF15-overexpressing construct and control vectors, respectively. The data are shown as the means ± SD (*n* = 3). *: compared with control group, *P < 0.05*. (**B**) Tumorigenicity of SK-SC-GDF15 and SK-SC-control cells. Cells (1 × 10^6^) were injected subcutaneously into the flanks of recipient NOD/SCID mice (*n* = 5). Animals were sacrificed when tumor nodules were identified on the body surface of mice. Tumor weight was weighed. The data are shown as the means ± SD. *: compared with control group, *P < 0.05*. (**C** and **D**) Pulmonary metastasis of SK-SC-GDF15 and SK-SC-control cells was monitored using bioluminescence imaging *in vivo*. The growth of pulmonary metastasis was observed using whole-body fluorescent imaging after 4 weeks of cell injection. The relative photon flux of the two groups was calculated and tumor number was measured. The data are shown as the means ± SD (*n* = 5). *: compared with control group, *P < 0.05*; **: compared with control group, *P < 0.01*. (**E**) Representative images of HE staining of lungs in both groups after 4 weeks of cell injection. Scale bar: 100 μm.

To confirm these results, we transfected SK-SCs with GDF15-overexpressing and control vectors (Figure 5A). Our studies demonstrated that tumor volume and weight in the GDF15 overexpression group were significantly higher than that of the control group (Figure 5B), and GDF15 overexpression significantly increased the lung metastasis of SK-SCs (Figure 5C–5E). Overall, these findings suggest that GDF15 promotes LCSC growth and metastasis.

### GDF15 knockdown inhibits AKT/GSK-3β/β-catenin signaling pathway in SCs

**Figure 6:**
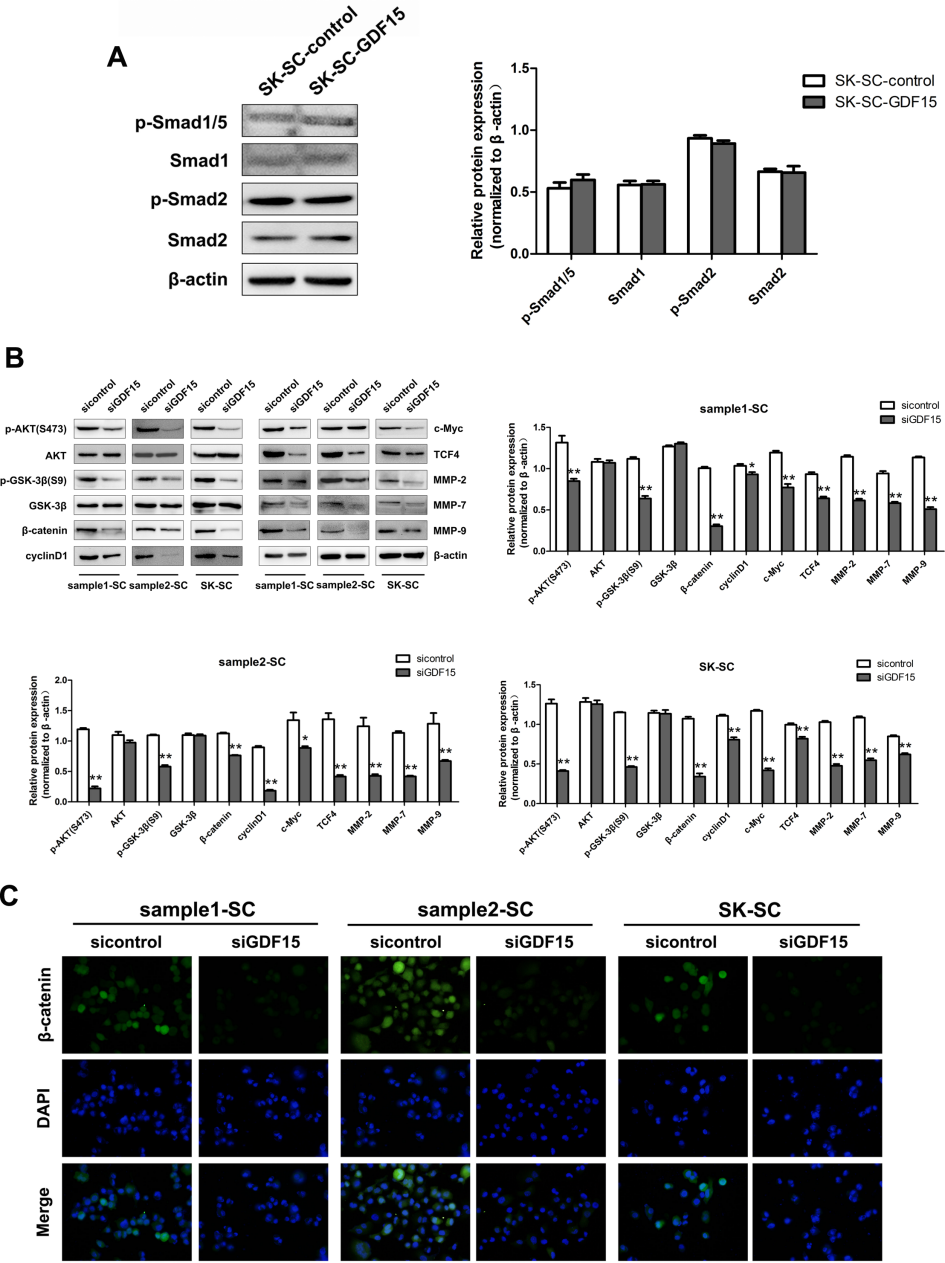
GDF15 knockdown inhibits AKT/GSK-3β/β-catenin signaling pathway in SCs (**A**) The total and phosphorylation levels of Smad1/5 and Smad2 in GDF15-overexpressing SK-SCs were detected by western blot analysis. Relative protein intensities were detected by Image J software. β-Actin was used as a loading control. (**B**) The main protein components of the AKT/GSK-3β/β-catenin signaling pathway in GDF15 knockdown SCs were measured by western blot analysis. Relative protein intensities were detected by Image J software. β-Actin was used as a loading control. *: compared with control group, *P < 0.05*; **: compared with control group, *P < 0.01*. (**C**) The expression of nuclear and cytoplasmic β-catenin in GDF15 knockdown SCs was determined by immunofluorescence assay.

To elucidate the underlying mechanism by which GDF15 regulates the proliferation and metastasis of SCs, several intracellular signaling pathways were measured in GDF15 overexpression or GDF15 knockdown SCs. As a TGF-β superfamily member, the GDF15 receptor is still relatively unknown today. Therefore, to confirm whether it could activate the TGF-β signal pathway, we detected the expression of Smad1, Smad2 and Smad5 in GDF15-overexpressing SK-SCs. The results showed that the phosphorylation levels of Smad1/5 and Smad2 were not altered by GDF15 (Figure 6A). The total protein and phosphorylated-Smad3 levels were not detectable in SK-SCs (data not shown).

However, we found that GDF15 knockdown significantly decreased the protein levels of p-AKT, p-GSK-3β and β-catenin in three groups of SCs and inhibited the expression of downstream molecules, such as cyclinD1, c-Myc, TCF4, MMP-2, MMP-7 and MMP-9 (Figure 6B). To further determine nuclear and cytoplasmic β-catenin levels in GDF15 knockdown SCs, we performed immunofluorescence (IFC) assay. The results showed that GDF15 knockdown decreased β-catenin expression (Figure 6C). These results indicate that the AKT/GSK-3β/β-catenin pathway is associated with inhibition of SC proliferation and metastasis by GDF15 knockdown.

### Blocking AKT/GSK-3β/β-catenin signaling pathway suppresses GDF15-driven proliferation, colony formation and invasion of SCs

**Figure 7:**
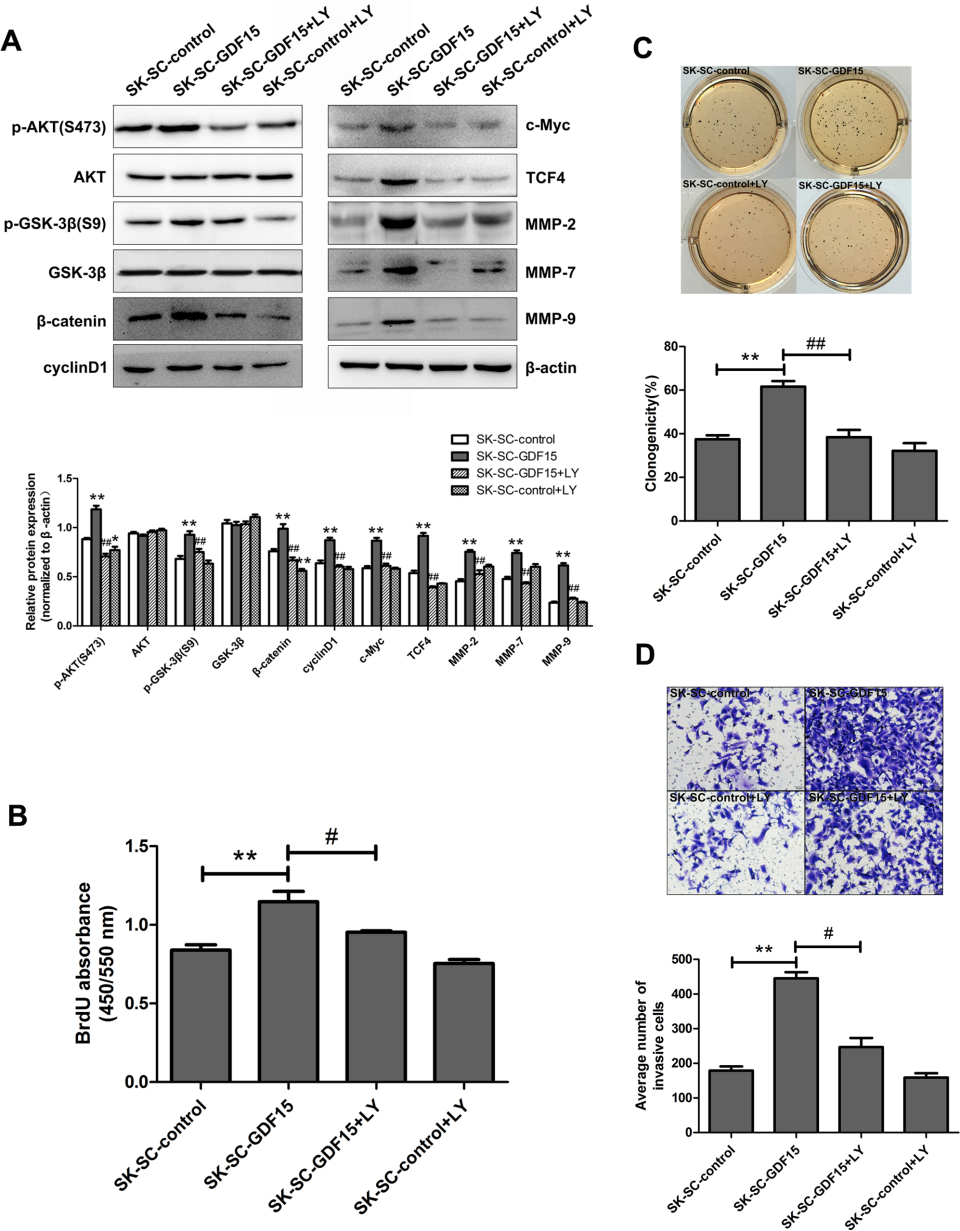
Blocking AKT/GSK-3β/β-catenin signaling pathway suppresses GDF15-driven proliferation, colony formation and invasion of SCs (**A**) GDF15-overexpressing SK-SCs were treated with 1 μM PI3K inhibitor LY294002, and the molecule expression levels of AKT/GSK-3β/β-catenin signaling were analyzed by western blot. Relative protein intensities were detected by Image J software. β-Actin was used as a loading control. *: compared with SK-SC-control group, *P < 0.05*; **: compared with SK-SC-control group, *P < 0.01*; ^##^: compared with SK-SC-GDF15 group, *P < 0.01*. LY294002 treatment significantly decreased the proliferation, colony formation and invasion ability of GDF15-overexpressing SK-SCs, as shown by BrdU assay (**B**), colony formation assay (**C**) and transwell assay (**D**). The data are shown as the means ± SD. Scale bar: 50 μm. **: compared with SK-SC-control group, *P < 0.01*; ^#^: compared with SK-SC-GDF15 group, *P < 0.05*; ^##^: compared with SK-SC-GDF15 group, *P < 0.01*.

To further determine the role of the AKT/GSK-3β/β-catenin signaling pathway in SC proliferation, and metastasis, GDF15-overexpressing SK-SCs were treated with the PI3K inhibitor LY294002. The results showed that GDF15 overexpression significantly increased the expression of p-AKT, p-GSK-3β, β-catenin, cyclinD1, c-Myc, TCF4, MMP-2, MMP-7 and MMP-9 (Figure 7A). After LY294002 was used to inhibit AKT, the expression of p-AKT, p-GSK-3β, β-catenin, cyclinD1, c-Myc, TCF4, MMP-2, MMP-7 and MMP-9 was significantly decreased in GDF15-overexpressing SK-SCs (Figure 7A). Meanwhile, the effects of AKT/GSK-3β/β-catenin signaling inhibition on GDF15-overexpressing SK-SCs’ proliferation, colony formation and invasion ability were evaluated by BrdU assay, colony formation assay and transwell assay. The results showed that AKT pathway inhibition suppressed SK-SCs proliferation (Figure 7B), colony formation (Figure 7C) and invasion (Figure 7D) induced by GDF15 overexpression. Taken together, these results confirm that GDF15 induces SCs proliferation and metastasis via activating AKT/GSK-3β/β-catenin signaling pathway.

## DISCUSSION

LCSCs are a small subpopulation in liver cancer, have been proposed to be cancer-initiating cells, and have a stronger migration ability and tumorigenicity that is responsible for chemotherapy resistance, cancer metastasis and recurrence. These findings indicate that liver cancer therapies may simply kill a majority of tumor cells without eradication of LCSCs, and the key to improving the curative effect of treatments for liver cancer is the eradication of LCSCs. Therefore, to achieve better understanding and targeting of LCSCs, we must further discover LCSC biomarkers and clarify cellular and signaling functions of LCSCs. In the present study, we attempted to isolate liver cancer stem-like cells derived from clinical HCC samples and hepatocarcinoma cell lines using sphere-forming culture. Cells growing under sphere-forming conditions show increased viability, suggesting stem cell characteristics of CSCs. We successfully established and cultured three groups of SCs from two HCC samples and SK-Hep-1 cells. All three groups of SCs exhibited the CSC-like properties of high tumorigenicity, high metastasis and strong resistance to chemotherapy.

Although many surface markers have been reported for LCSCs, such as CD13, CD44, CD133 and EpCAM, there is still unified standard [13]. LCSC surface markers need to constantly being discovered, and their sensitivity and specificity require further study. In the present study, our results revealed that SCs derived from two human HCC samples and SK-Hep-1 cells, were characterized by co-expression of CD13 and CD44. CD13, a membrane-bound zinc-dependent type II exopeptidase, is highly expressed in many tumor cells and plays a crucial role in tumor growth, metastasis and angiogenesis. Haraguchi et al. [14] indicated that CD13 was a marker for semiquiescent CSCs in human liver cancer cell lines and clinical samples and that targeting these cells might provide a way to treat HCC. Meanwhile, CD44 is also an important marker in HCC. CD44 and other markers were reported to more accurately define the phenotype of LCSCs [15, 16]. In agreement with these previous reports, our findings suggest that CD13^+^CD44^+^ may be a specific marker of these SCs. Although CD133 and EpCAM are commonly considered as the LCSC markers^4^, the expression of these factors was in a very low level in the isolated SCs. Based on these results, we speculate that the CSC phenotype may be different in each HCC subtype, possibly due to the heterogeneity of activated signaling pathways in normal stem cells where these cancer stem cells could originate. The expression patterns of various CSC markers may represent a subset of LCSCs. Therefore, our studies demonstrate that CD13^+^CD44^+^ SCs may represent a subset of LCSCs and display the capacity for promoting tumor growth, invasion and metastasis.

Recent findings suggest that LCSCs initiate and maintain HCC growth and translocation from the primary tumor to distant tissues, leading to new tumors [17]. However, the underlying biological mechanism of LCSCs in HCC recurrence and metastasis is unclear. According to the results of cytokine antibody array, we found that GDF15 expression was significantly increased in SCs. In addition, GDF15 was overexpressed in a part of HCC patients and was positively related to pathological grading of HCC. GDF15 is a member of the TGF-β superfamily. GDF15 is not only involved in cancer development, progression, angiogenesis and metastasis, but also controls embryonic, osteogenic and hematopoietic development, stress responses, adipose tissue function and cardiovascular diseases [18]. So far, few studies have elucidated the relationship between GDF15 and hepatocarcinoma. Especially, the biological significance of GDF15 in LCSC properties and HCC pathogenesis remains uncertain. Researchers investigated the effect of GDF15 loss *in vivo* on hepatocellular carcinogenesis and found that genetic ablation of GDF15 had no apparent effect on the tumor formation, growth or invasiveness in a diethylnitrosamine-induced HCC mouse model [19]. However, our results indicated that GDF15 knockdown suppressed the proliferation and colony formation of SCs *in vitro* and attenuated SCs tumorigenesis *in vivo*, suggesting that GDF15 enhanced tumorigenicity in SCs. Our results also demonstrated that GDF15 knockdown inhibited the invasive and metastatic properties of SCs, whereas GDF15 overexpression promoted SC metastatic capability. These results were consistent with previous reports which indicated that GDF15 overexpression enhanced the proliferation and invasiveness of HCC cell line Huh7.5.1 [20] and human breast cancer cell line SK-BR-3 [21]. These findings suggest that GDF15 contributes to the carcinogenesis of HCC and promotes the growth and metastasis of LCSCs. Moreover, these opposite effects of GDF15 on HCC progression may be dependent on the stage and type of HCC.

The receptor and signaling pathways of GDF15 have not been unequivocally identified, although several of its biological functions have already been described. Several studies have suggested that GDF15 might mediate certain cellular responses through a Smad-dependent pathway or a Smad-independent pathway. It had been reported that GDF15 stimulated ovarian cancer cell growth and invasion through AKT/mTOR and MAPK signaling [22]. A recent study indicated that GDF15 promoted EMT and metastasis in colorectal cancer through binding to the TGF-β receptor to activate Smad2 and Smad3 pathways [23]. In the current study, we did not find that Smad signaling was activated by GDF15 in SK-SCs, suggesting that GDF15-mediated growth and metastasis might be TGF-β receptor-independent, at least for SK-SCs.

However, we next identified phosphorylated AKT as the downstream component of GDF15-mediated signaling in SCs. The contribution of the AKT pathway to HCC growth and metastasis has been extensively studied [24, 25]. Our data indicated that GDF15 induced AKT phosphorylation in SCs, which was consistent with the observation from Kim et al [26], who found that GDF15 activated AKT signaling in human breast and gastric cancer. The aberrant Wnt pathway is critically important in CSC biology. The interaction between the two pathways through the AKT/GSK-3β/β-catenin axis is found to participate in the maintenance of CSC properties [27, 28]. In the AKT/GSK-3β/β-catenin signaling pathway, GSK-3β plays an important role in β-catenin phosphorylation and degradation. GSK-3β activity can be inhibited by AKT phosphorylation at Ser9 and β-catenin transfers into the nucleus to activate the transcription of downstream target oncogenes such as cyclin D1, c-Myc, MMP-2 and MMP- 7. In our study, GDF15 knockdown reduced β-catenin levels through AKT/GSK-3β signaling suppression, and inhibited the expression of β-catenin-responsive cyclinD1, c-Myc, TCF4, MMP-2, MMP- 7 and MMP-9 genes, resulting in the suppression of LCSC properties. Moreover, a PI3K inhibitor LY294002 significantly inhibited AKT/GSK-3β/β-catenin signaling activated by GDF15 and attenuated GDF15-induced proliferation, colony formation and invasion of SCs. The current studies suggest that the molecular mechanism of inducing proliferation and metastasis by GDF15 may depend on the activation of the AKT/GSK-3β/β-catenin signaling pathway.

In conclusion, CD13^+^CD44^+^ SCs may represent a subset of LCSCs, which exhibit a strong CSC-like potential for tumorigenesis and metastasis. Our studies highlight a novel mechanism suggesting that GDF15 may promote the growth and metastasis of SCs by activating the AKT/GSK-3β/β-catenin pathway. This work is of particular significance in suggesting that GDF15-AKT/GSK-3β/β-catenin signaling is a novel mechanism driving LCSC growth and metastasis. GDF15-targeted approaches should be further investigated as potential therapeutic strategies for HCC.

## MATERIALS AND METHODS

### Tissue samples

HCC tissues and PL tissues were collected from Chinese patients with hepatocarcinoma treated with curative liver resection at the Cancer Institute and Hospital of Tianjin Medical University. This investigation was supported by the Ethics Committee of Cancer Institute and Hospital of Tianjin Medical University. All the patients provided written informed consent for the use of tumor tissue in clinical and basic research.

### Cell culture and reagents

HCC cell lines SK-Hep-1 and Hep3B were obtained from the American Type Culture Collection (ATCC). HCC cell lines Bel-7402 and SMMC-7721 were provided by the Cell Bank of Institute of Biochemistry and Cell Biology, China Academy of Sciences (Shanghai, China). All cell lines were cultured in Dulbecco’s modified Eagle’s medium (GIBCO, USA) containing 10% heat-inactivated fetal bovine serum (GIBCO, USA). The following antibodies were used for flow cytometry: anti-CD44-FITC, FITC-labeled affinity-purified antibody to mouse IgG1, anti-EpCAM-FITC and FITC-labeled affinity-purified antibody to mouse IgG2b (Biolegend, USA); anti-CD133-APC, APC-labeled affinity-purified antibody to mouse IgG2b (Miltenyi Biotec, CA); anti-CD13-APC, and APC-labeled affinity-purified antibody to mouse IgG1 (eBioscience, USA). The following antibodies were used for western blot: Oct4 (1:500, Upstate, USA), Nanog (1:500, Upstate, USA), GDF15 (1:1000, Abcam, USA), AKT (1:1000, Cell Signaling Technology, USA), p-AKT(S473) (1:1000, Cell Signaling Technology, USA), GSK-3β (1:1000, Cell Signaling Technology, USA), p-GSK-3β(S9) (1:1000, Cell Signaling Technology, USA), β-catenin (1:1000, Cell Signaling Technology, USA), cyclinD1 (1:1000, Cell Signaling Technology, USA), c-Myc (1:1000, Cell Signaling Technology, USA), TCF4 (1:1000, Cell Signaling Technology, USA), MMP-2 (1:500, Santa Cruz, USA), MMP-7 (1:500, Boster, China), MMP-9 (1:500, Boster, China), Smad1 (1:1000, Cell Signaling Technology, USA), p-Smad1/5 (1:1000, Cell Signaling Technology, USA), Smad2 (1:1000, Cell Signaling Technology, USA), p-Smad2 (1:1000, Cell Signaling Technology, USA) and β-actin (1:12000, Sigma, USA). Specific PI3K inhibitor LY294002 was obtained from Selleck Chemicals (USA). HCC tissue array was obtained from Biomax (USA).

### Isolation and culture of tumor spheres

HCC tissues were minced and digested with 0.15% type IV collagenase (Sigma, MO). Cell suspension was passed through a 40 μM filter. After lysis of red blood cells, cells were cultured in SFM composed of DMEM/F12 (1:1) medium (Hyclone, USA) supplemented with 20 ng/ml EGF (PeproTech, USA), 10 ng/ml bFGF (PeproTech, USA), 5 ng/ml SCF (PeproTech, USA), 2 ng/ ml LIF (Millipore, USA) and 2% B27 (GIBCO, USA). The SK-Hep-1, Hep3B, Bel-7402 and SMMC-7721 cell lines were trypsinized and cultured in SFM. Morphological changes in the tumor cells were observed under an inverted light microscope.

### Cell proliferation assay, cell cycle analysis and drug sensitivity assay

Cell proliferation was measured by BrdU assay (Millipore, USA). For cell cycle analysis, after fixation in 70% ethanol, cells were incubated in PI/RNase (BD Biosciences, USA), and analyzed using an Accuri C6 flow cytometer and ModFit LT software (BD Biosciences, USA). For drug sensitivity assay, cells were treated with 0.4 mg/ml 5-FU, 0.004 mg/ml DDP and 0.0004 mg/ml ADM (sigma, USA) for 48 hours. MTS reagent (Sigma, USA) was added to each well following the manufacturer’s instructions to determine drug sensitivity.

### Colony formation assay

Cells were suspended in an appropriate medium with 0.3% agarose and plated in 12-well plates covered with suspension medium supplemented with 0.5% agarose, at a density of 500 cells per well. After 2 weeks, colonies were dyed with MTT (Sigma, USA) and counted under the microscope.

### Invasion assay

Cells were seeded at a density of 5 × 10^4^ cells into the upper chamber of each insert coated with Matrigel (BD Biosciences, USA). The medium containing 20% FBS was added to the lower chambers as a chemoattractant. After incubation for 48 hours, cells invading the lower surface of the filter were fixed with 4% paraformaldehyde and stained with crystal violet. Cell numbers were counted in three random fields per filter.

### *In vivo* tumorigenicity and lung metastasis

Five-week-old female NOD/SCID mice were purchased from the Animal Institute of Peking Union Medical College. *In vivo* tumorigenicity experiments were conducted by injecting various cells subcutaneously into NOD/SCID mice. The experiments were terminated when tumor nodules were identified on the body surface of mice. *In vivo* models of lung metastasis were created by injecting the transducing cells with lentiviral vectors expressing luciferase into NOD/SCID mice via the tail vein. Lung metastatic colonization was monitored and quantified at different weeks with bioluminescence imaging using an IVIS Spectrum *in vivo* imaging system (PerkinElmer, Waltham, MA), and validated at the endpoint by hematoxylin-eosin (HE) staining. Procedures in these experiments were approved by the Institutional Animal Care and Use Committee at Tianjin Medical University.

### Cytokine antibody array

SK-SCs and SK-Hep-1 Cells were seeded in 100 mm culture dishes and incubated for 48 hours. Cell culture supernatants were analyzed for protein expression using a RayBio^®^ L-Series Human Antibody Array 1000 Glass Slide Kit (RayBiotech, USA), according to the manufacturer’s instructions. The images were captured using an Axon GenePix laser scanner.

### ELISA

Human GDF15 immunoassay (R&D systems, USA) was conducted according to the manufacture’s directions. Optical density was determined using a microplate reader set to 450 nm. The concentrations were calculated according to the standards.

### Plasmids and GDF15 transfection

The GDF15 shRNA target sequence was 5′-TCTCAGATGCTCCTGGTGTTG-3′. A lentiviral pSUPER-GFP vector was purchased from OligoEngine (USA). Lentiviral helper plasmids (PMDL, VSVG and RSV-REV) were obtained from Addgene (Biovector Inc, USA). GDF15-overexpressing lentivirus was obtained from Shanghai Genechem Co., Ltd. Virus supernatant was incubated on target cells for 12 hours with 5 μg/ml polybrene, following the manufacturer’s instructions. Infected cells were selected in puromycin, as optimized for each cell line.

### RNA isolation and RT-PCR

Total RNA was isolated using Trizol reagent (Invitrogen, USA). Total RNA (2 μg) was used for the synthesis of first-strand cDNA using M-MLV reverse transcriptase (Invitrogen, China). Quantitative real-time PCR was performed using the SYBR green mix (Applied Biosystems, USA). The reactions were performed with a 7900 Fast Real-Time PCR System (Applied Biosystems, USA). The data were displayed as 2^–ΔCt^ values and were representative of at least three independent experiments. Specific primers for the amplification of target genes and β-actin, a housekeeping gene, are listed in Supplementary Table 1.

### Protein extraction and western blot analysis

The protein concentration of cell extracts was determined using the BCA Protein Assay Kit (Pierce, USA). Western blot analysis was performed as previously described [23]. Antibody binding was revealed using an HRP-conjugated anti-rabbit IgG or anti-mouse IgG (Sigma, USA). Antibody complexes were detected using Immobilon Western Chemiluminescent HRP Substrate (Millopore, USA).

### Immunohistochemistry and immunofluorescence staining

The IHC and IFC staining were performed as previously described [29, 30]. The antibodies used for immunostaining were GDF15 (1:100, Abcam, USA) and β-catenin (1:100, Cell Signaling Technology, USA).

### Statistical analysis

Each experiment was performed in triplicate and repeated at least three times. Data were presented as mean ± standard deviation (SD). The statistical analyses were conducted with SPSS 20.0. The criterion for statistical significance was set at *P < 0.05*.

## SUPPLEMENTARY MATERIALS TABLE AND FIGURES



## Abbreviations

HCC: hepatocellular carcinoma
CSCs: cancer stem cells
EMT: epithelial-mesenchymal transition
LCSCs: liver cancer stem cells
SCs: sphere cells
SFM: serum-free medium
MDR1: multidrug resistance gene-1
MRP1: multidrug resistance associated proteins 1
BCRP: breast cancer resistance protein
CM: culture medium
IHC: immunohistochemistry
PL: paracarcinomatous liver
IFC: immunofluorescence.
