# Supplementary material for: Growth differentiation factor 15 induces growth and metastasis of human liver cancer stem-like cells via AKT/GSK-3β/β-catenin signaling
Source: Oncotarget. 2017 Feb 9;8(10):16972–87. doi: 10.18632/oncotarget.15216 (PMC5370015; doi:10.18632/oncotarget.15216)
Supplement: Supplementary file 1 [file oncotarget-08-16972-s001.pdf]

Growth differentiation factor 15 induces growth and metastasis of human liver cancer stem-like cells via AKT/GSK-3β/β-catenin signaling

Supplementary Materials

Supplementary Table 1: Primers used for RT-PCR analysis

Genes	Forward primer	Reverse primer
MDR1	5′-AGACATGACCAGGTATGCCTAT-3′	5′-AGCCTATCTCCTGTCGCATTA-3′
MRP1	5′-GTCGGAACAAGTCGTGCCTG-3′	5′-CAAAGCCTCCACCTCCTCATTC-3′
ABCG2	5′-TGGCTGTCATGGCTTCAGTA-3′	5′-GCCACGTGATTCCACAA-3′
β-actin	5′-TTGCCGACAGGATGCAGAAGGA-3′	5′-AGGTGGACAGCGAGGCCAGGAT-3′

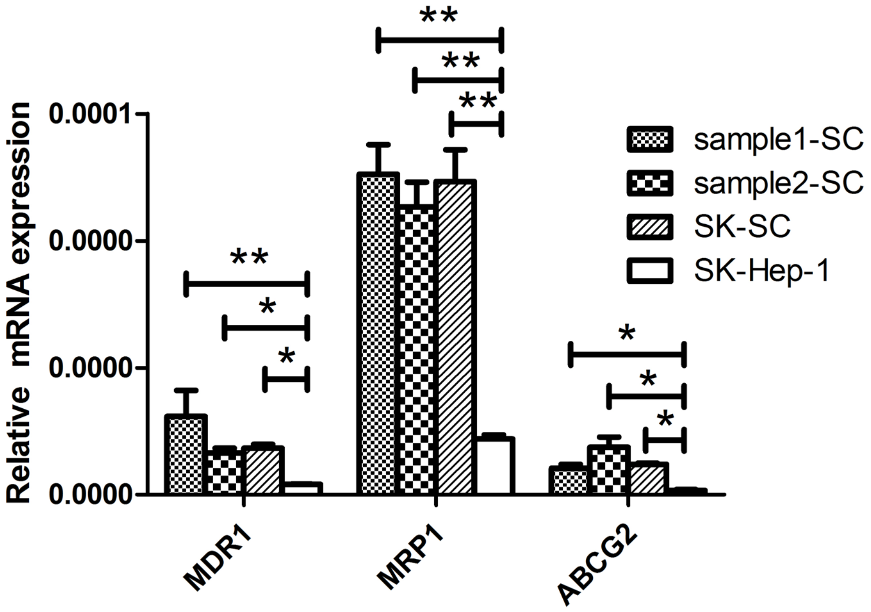

Supplementary Figure 1: ABC transporters such as MDR1, MRP1 and ABCG2 are highly expressed in three groups of SCs. Expression of MDR1, MRP1 and ABCG2 in SCs and SK-Hep-1 cells were detected by RT-PCR. β-Actin was used as an internal reference. Values represent the average expression in normalized transcript ($n = 3$). *: compared with SK-Hep-1 cells, $P < 0.05$; **: compared with SK-Hep-1 cells, $P < 0.01$.

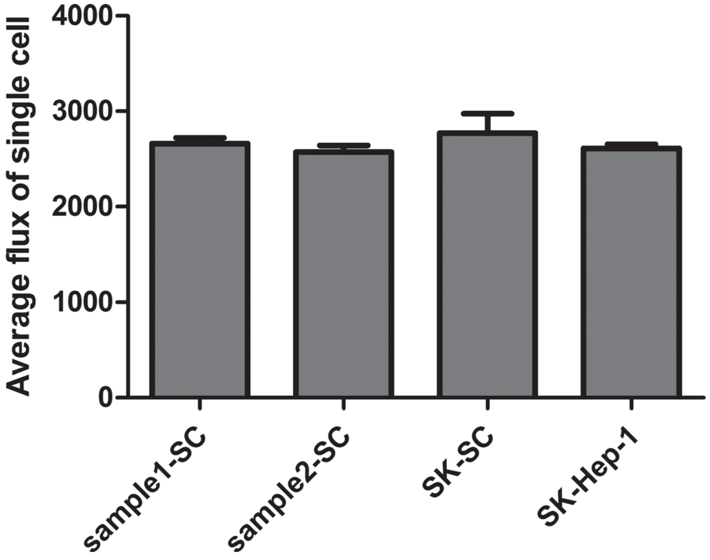

Supplementary Figure 2: The mean fluorescence intensities of the single SC and SK-Hep-1 cell transduced with luciferase-expressing lentiviral vectors are similar. Three groups of SCs and SK-Hep-1 cells were transduced with luciferase-expressing lentiviral vectors. The mean fluorescence intensities of the luciferase-expressing SCs and SK-Hep-1 cells were detected by bioluminescence imaging. Values represent the average expression in normalized photon flux (n = 3).

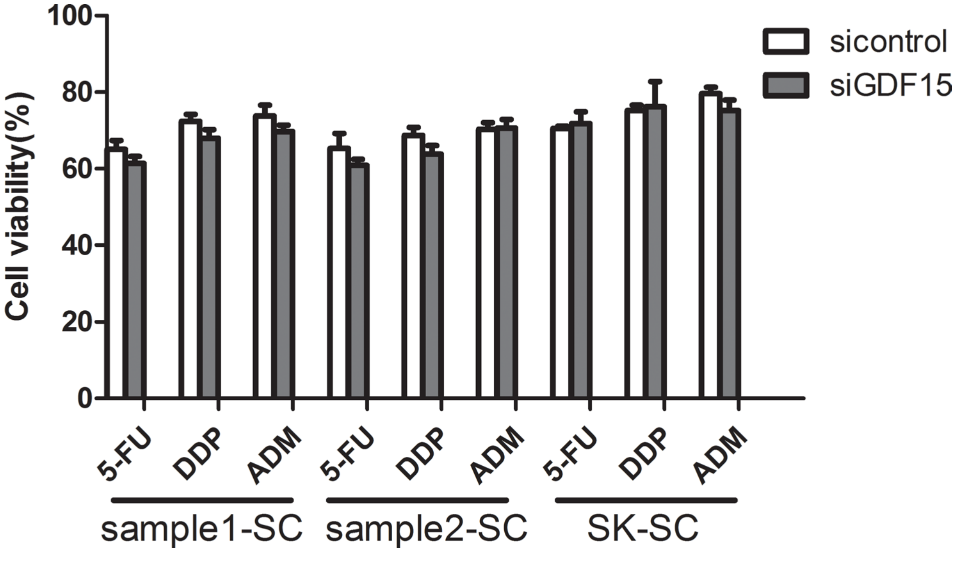

Supplementary Figure 3: GDF15 has no effect on drug resistance of SCs. GDF15 knockdown SCs were treated with 5-FU, DDP and ADM for 48 hours. The cell viability was measured with MTS and calculated using the following formula: Cell viability (%) = (absorbance of treated cells)/(absorbance of untreated cells) ×100%. The data are shown as the means ± SD (n = 3).
